# A Simple and Low-cost Method of Sleeve to Insert Silicone Gel Breast Implants

**DOI:** 10.1097/GOX.0000000000002389

**Published:** 2019-09-30

**Authors:** Georg Panczel, Alexandre Mendonça Munhoz

**Affiliations:** From the * Department of Plastic Surgery, Aleris Stavanger Private Hospital, Stavanger, Norway; † Department of Hand and Plastic Surgery, Stavanger University Hospital, Stavanger, Norway; ‡ Plastic Surgery Division, Hospital Sírio-Libanês, São Paulo, Brazil; § Plastic Surgery Division, University of São Paulo School of Medicine, São Paulo, Brazil; ¶ Plastic Surgery Department, Hospital Moriah, São Paulo, Brazil.

## Abstract

Contact between silicone implants and skin/breast parenchyma has been described as an agent of implant contamination and biofilm formation, resulting in implant complications. The no-touch technique was introduced to avoid implant contact and reduce skin/breast contamination. The authors propose an easily available sleeve option using a saline sterile plastic container that provides elastic and transparent protection for inserting silicone implants. These sterile containers can be easily converted into a sleeve by cutting off the narrow end of the container, which then easily fits into the small inframammary, periareolar, and transaxillary incisions. The authors have performed this technique in 10 patients (20 implants) undergoing primary breast augmentation or revision breast surgery, with microtexturized implants ranging in size from 255 to 500 ml (mean, 325 ml) and obtained satisfactory results with no cases of complications. Our clinical outcome shows that this new sleeve does not interact with the implant or the patient’s skin and soft tissues and has the added advantage of low cost compared with similar available devices, but further randomized and controlled studies are required to corroborate this effect.

## INTRODUCTION

Breast augmentation with silicone gel implants (SGIs) is one of the most common esthetic breast surgery procedures^1,2^; since the first introduction of these implants in the 1960s, breast augmentation techniques have progressed.^3^ Contact between skin and SGI is described as an agent of implant contamination and subclinical infection,^4^ and biofilms have also been associated with SGI complications including capsular contracture (CC)^5,6^ and double capsule.^7^ More recently, the infectious theory related to bacterial contamination has been indicated as one etiology of breast implant–associated anaplastic large-cell lymphoma (BI-ALCL).^8,9^

To avoid contact between SGI and skin/parenchyma, Dolsky^10^ first introduced the concept of an implant conduit made of polyethylene tubing to insert the Même implant. In the 1990s, a “no-touch technique” was described by Mladick^11^ in submuscular saline breast augmentation, based on retractors to hold the incision open and avoid skin contact. More recently, many plastic surgeons consequently advocate a no-touch technique to minimize skin/parenchyma contamination and complications.^9–14^ This is generally accomplished with a device composed of a nylon sleeve with a hydrophilic inner coating.^12^

This present article describes a low-cost sleeve adapted to insert smooth, nanotextured, and texturized SGI. The authors propose an easily available option: a sterile plastic saline container that is elastic, transparent, and protects the SGI during insertion.

## MATERIALS AND METHODS

**Fig. 1.**
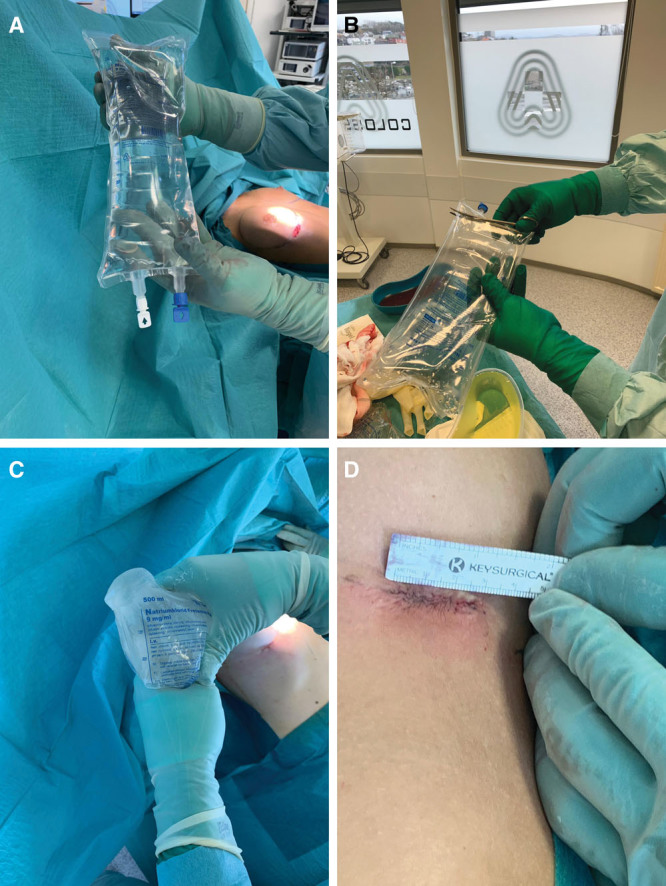
The insertion sleeve technique. A, The Freeflex container utilized as a sleever. The container is a non-PVC bag composed of several polyolefin layers and manufactured by polymerizing ethylene and propylene or copolymerizing these 2 monomers. B, The Freeflex container is easily converted into a sleeve by cutting off the closed, narrow end of the bag. C, The inside of the Freeflex bag is soaked with sterile saline or sterile water–soluble lubricant, which lubricates the surface and the SGI easily fits into the sleeve. D, Final result of the inframammary scar following the implant insertion using the no-touch technique was associated with the low-cost sleeve.

To insert SGI in breast augmentation and revision procedures, we have used infusion containers available in 500 and 1,000 cc sizes. The Freeflex container (Fresenius Kabi, Pymble, New South Wales, Australia) is a non-PVC bag composed of several polyolefin layers (polymers made from hydrogen and carbon atoms).^15^ They are manufactured by polymerizing ethylene and propylene or copolymerizing these 2 monomers (Fig. 1A). This bag was developed and tested to meet all the mechanical requirements of the third edition of *European Pharmacopoeia* for “plastic containers for aqueous solutions in parenteral infusion.”^15^ In our experience, this container can be easily converted into a sleeve by cutting off the closed, narrow end of the bag (Fig. 1B). This narrow end easily fits into small inframammary, periareolar, and transaxillary incisions. The inside of the bag is soaked with sterile saline or sterile water–soluble lubricant, which lubricates the surface and the SGI easily fits into the sleeve (Fig. 1C). Routinely, we followed 13 of the 14 points described by Adams et al,^9^ except pocket irrigation with betadine triple-antibiotic solution. After the incision is made and the pocket dissected, the surgeon places the narrow end of the sleeve into the pocket and inserts the SGI without any skin contact. The sleeve is then twisted so that the solution is squeezed inside the pocket while the sleeve itself is pulled back and easily removed (See Video [online], which displays the low-cost sleeve technique). We try as far as possible to perform the “no-touch technique” and adjust the implant inside the pocket with instruments. However, in rare situations, minor adjustments are necessary using fingers. In this specific situation, we perform the exchange of gloves and avoid contact of the finger with the skin. The skin is then finally closed by layer (Fig. 1D).

**Video.** This Video displays the low-cost sleeve technique.

## RESULTS

The authors have performed this technique in 10 patients (20 SGIs) undergoing primary augmentation or revision breast surgery. The implant ranged from 255 to 500 cc (mean, 325 cc), all microtexturized (Mentor Implants; Mentor, Irvine, Calif.). So far, no complications have been observed, and no implants have ruptured since this sleeve technique was adopted. There is a significant cost reduction in comparison with traditional products. In Norway (March 2019), 20 Freeflex bags cost an average of approximately US$ 34.00 (US$ 1.60 per unit). In contrast, the traditional funnel costs approximately US$ 85 per unit.

## DISCUSSION

Sleeves have been used for a no-touch SGI delivery device since the 1990s.^10–14^ The main advantages are reduced contamination risk, low-friction insertion with less force, and less risk of implant rupture.^11,12^ The other devices available on the market include the Keller Funnel (Keller Medical, Stuart, Fla.), which consists of a rip-stop nylon sleeve with a hydrophilic inner coating.^12^ It is described as a simple, predictable system and significantly decreases skin contact and bacterial contamination of SGI. Moyer et al^12^ assessed the Keller Funnel device with digital insertion in a cadaver model; these authors found that the funnel decreased skin contact by 27 times for all smooth SGI (*P* = 0.00059), and that bacterial contamination from breast parenchyma was 2 times more likely with the standard digital insertion technique (*P* = 0.06), indicating that this tool appears to significantly reduce skin contact and parenchyma contamination.^12^

We have found that this new sleeve is an elastic surface which provides enough protection from extrinsic mechanical forces and avoids potential SGI contamination. Based on our intraoperative experience and clinical outcomes, the sleeve does not interact with the SGI or the patient’s skin or soft tissues. The cut end of the plastic sleeve is not sharp and thus does not damage the implant. Furthermore, it is more cost-effective, readily available in any hospital or operating suite, quick to apply, and offers practical and economic advantages over the available alternatives.

Concerning the regulatory process, all medical devices sold in Norway and Brazil are regulated by the local regulatory agencies, as occurs in the United States with the Food and Drug Administration. Usually, regulatory policy for these medical devices follows a 3-tiered classification system: class I, II, and III. The higher numbered class, the greater the regulatory control, which further defines the regulatory requirements for a general device type. Class I medical devices are those that have a low to moderate risk to the patient and/or user and examples include elastic bandages, stethoscopes, and surgical sleeves already approved in the recent past. In our study, we used the Freeflex container (Fresenius Kabi, Pymble, New South Wales, Australia), a non-PVC bag composed of several polyolefin layers, and already approved as a class II medical devices as a saline solution container. This bag was developed and tested to meet all the mechanical requirements of the third edition of *European Pharmacopoeia* for plastic containers for aqueous solutions in parenteral infusion.

In our preliminary experience, we have not yet standardized the relationship between the sleeve opening and implant size. However, with greater experience and using different implant types and volumes, this standardization can be performed in further investigations. Up to now, we suggest to use straight blade scissors to trim the distal end of the sleeve. The Freeflex bag opening should be sized large enough to allow the implant to smoothly pass through the sleeve without damaging the implant and without being too large thus unintentionally enabling the implant to pass through and have a direct contact with the skin.

Despite the positive outcomes we have observed in our initial cases, one limitation of this study might be the short follow-up period, which may not provide enough time to evaluate rates of long-term infection, capsular contracture, double capsule, and BI-ALCL. No cases of infection were seen in a short-term evaluation, but future assessment with a larger sample and longer follow-up would provide more definitive conclusions. Therefore, additional randomized controlled studies that compare the different sleeve brands associated with cost analysis and long-term outcome will be beneficial to corroborate these observations. Similar observations can be made about the experience with only one type of implant. Thus, future studies comparing different implants, surfaces, and shapes would be necessary to evaluate the reproducibility of this low-cost sleeve. At the present moment, the main objective of this study is to present the new technique as “Ideas and Innovations.” We continue to use the technique with good results and follow the patients regarding the main complications. In the near future, these data will be published in a new study with a large sample, different implants, and longer follow-up.

Up to this point, our impression is that this new sleeve allows the surgeon to perform breast augmentation/revision with microtexturized implants using the no-touch technique, in a simple, reproducible, and in a more economical manner.
